# Age and spatial effects of *Eimeria* spp. infections in European hare (*Lepus europaeus*) killed by vehicle collisions

**DOI:** 10.1017/S0031182025100735

**Published:** 2025-09

**Authors:** Jan Hušek, Kateřina Brynychová, Jan Cukor, Jakub Hruška, Jana Kvičerová

**Affiliations:** 1 Department of Zoology, National Museum of the Czech Republic, Prague, Czech Republic; 2 Department of Biology, Faculty of Science, University of Hradec Králové, Hradec Králové, Czech Republic; 3 Forestry and Game Management Research Institute, Jíloviště, Czech Republic; 4 Faculty of Forestry and Wood Sciences, Czech University of Life Sciences Prague, Prague, Czech Republic; 5 Czech Geological Survey, Prague, Czech Republic; 6 Global Change Research Institute, Czech Academy of Sciences, Brno, Czech Republic; 7 Department of Parasitology, Faculty of Science, University of South Bohemia in České Budějovice, České Budějovice, Czech Republic; 8 Department of Zoology, Faculty of Science, Charles University, Biocev, Vestec, Czech Republic

**Keywords:** behaviour, coccidiosis, epidemiology, lagomorph, parasite, prevalence, road mortality, wildlife

## Abstract

Risk factors for *Eimeria* infections are well documented in farm and pet animals, but studies focusing on wildlife species are less common. This research aimed to investigate the impact of selected demographic and environmental factors on the prevalence of *Eimeria* in the European hare (*Lepus europaeus*). Additionally, we analysed whether *Eimeria* infection affected the behaviour of hares by examining the relationship between infection status and the likelihood of a hare being killed by a vehicle at a hotspot for road mortality. Between 11 February 2022 and 24 June 2024, we collected 22 hare carcasses that had been killed in traffic along an 83.9 km monitoring route in central Bohemia, Czech Republic, to evaluate *Eimeria* prevalence in relation to factors such as age, hare density, distance to the nearest water source and rainfall over the previous 3 months. Contrary to our expectations, we found a higher prevalence of *Eimeria* in adult hares compared to juveniles. We propose that this outcome may be due to the high mortality rates among leverets and juvenile hares, which removes susceptible individuals from the population early on. The effects of the other factors examined were not significant. In conclusion, our study revealed that *Eimeria* infection did not contribute to the clustering of hare–vehicle collisions. We emphasize the importance of studying risk factors in wildlife species across different ecological contexts. Our findings challenge the general assumption that age negatively influences *Eimeria* prevalence.

## Graphical abstract

**Figure:**
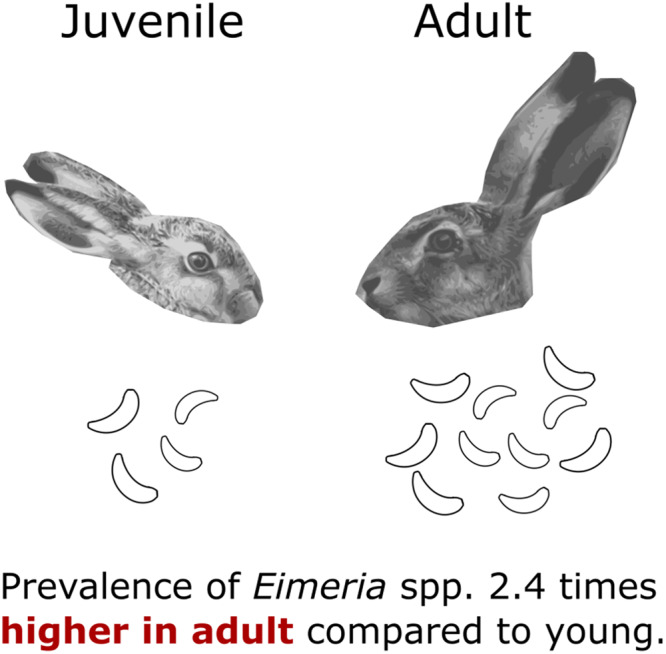

## Introduction

Parasites of the genus *Eimeria* (Apicomplexa: Coccidia) may cause severe disease (coccidiosis) in a wide range of domestic and wildlife species, including leporids (Pakandl, 2009; Duszynski and Couch, 2013; Duszynski and Morrow, 2014; Duszynski et al., 2018; Mesa-Pineda et al., 2021; Bangoura et al., 2022). The life cycle of *Eimeria*, its inter-population and seasonal variability, and major risk factors have been well described in many host species, with a focus mainly on farm and domestic animals (Pakandl, 2009; Geng et al., 2021; Mesa-Pineda et al., 2021; Bangoura et al., 2022). Due to the mode of their transmission and dispersal, *Eimeria* infections typically manifest as an emergent disease, affecting local populations rather than individuals (Mesa-Pineda et al., 2021; Bangoura et al., 2022).

Limited movements and high population densities may promote the spreading of the infection at a fast rate (Geng et al., 2021; Mesa-Pineda et al., 2021; Bangoura et al., 2022). Furthermore, weather conditions are among the most important risk factors of infection in farm animals kept under extensive farming regimes and wildlife species (Bangoura et al., 2022). For example, the prevalence of bovine *Eimeria* infections in tropical areas is higher in the rainy compared to the dry season and increases with the average temperature (Rodríguez-Vivas et al., 1996; Pfukenyi et al., 2007; Makau et al., 2017). In a French population of wild rabbits (*Oryctolagus cuniculus*), the prevalence was higher in the humid and relatively cold areas compared to the drier and warmer ones during 1998–1999 (Grès et al., 2003). In Australian wild rabbits, the prevalence was affected by rainfall and evaporation (Stodart, 1968).

While the prevalence of *Eimeria* and oocyst excretion exhibits clear annual seasonality in ruminants (Rodríguez-Vivas et al., 1996; Pfukenyi et al., 2007; Rehman et al., 2012), there is a strong intra- and interannual variation in lagomorphs (Stodart, 1968). In wild rabbits (*O. cuniculus*), different *Eimeria* species reach the highest prevalences at various times of the year (Grès et al., 2003; Foronda et al., 2005). Unlike the number of excreted oocysts, *Eimeria* prevalence in European hare (*Lepus europaeus*) did not show any seasonality from November to April in a study from the Czech Republic (Lukešová et al., 2012).

Indeed, there is a considerable large-scale variation among European hare populations in the composition of *Eimeria* parasites and their prevalences. The number of so far described *Eimeria* species found parasitizing local populations ranges from 4 to 16, they differ in pathogenicity and the total prevalence ranges from 20% to 100% across Central Europe (Forstner and Ilg, 1982; Chroust, 1984; Böckeler et al., 1994; Wibbelt and Frölich, 2005; Dubinský et al., 2010; Chroust et al., 2012; Lukešová et al., 2012; Kornaś et al., 2014; Brustenga et al., 2023; Faehndrich et al., 2023).

Development of partial or even full immunity in adults has been discussed for both mammals and birds (Tellez et al., 2014; Mesa-Pineda et al., 2021; Bangoura et al., 2022; Sîrbu et al., 2022). Immunization has been considered as an explanation for the general reduction of *Eimeria* infections in older individuals compared to young ones across species, countries and ecological settings (Stodart, 1968; Grès et al., 2003; Pfukenyi et al., 2007; Lassen et al., 2009; Bangoura et al., 2012; Chroust et al., 2012; Rehman et al., 2012; Kornaś et al., 2014; Makau et al., 2017; Faehndrich et al., 2023). *Eimeria* spp.’s higher prevalence in young animals than in adults has also been reported in European hares from southern Poland (Kornaś et al., 2014). The only exception reported so far is Soay sheep (*Ovis aries*) from Hirta, St. Kilda, in which the prevalence of *Eimeria granulosa* increased with age (Craig et al., 2007).

We studied the effects of selected risk factors on the prevalence of *Eimeria* in European hares, a species that has experienced a severe long-term population decline across Europe since the second half of the 20th century caused by habitat and climate change, increase in predation pressure and possibly by pathogenic factors (Smith et al., 2005; Panek et al., 2006; Dubinský et al., 2010). Surprisingly, drivers of *Eimeria* prevalence have been rarely addressed in populations of wild hare (Kornaś et al., 2014), despite coccidiosis being recognized as an important source of their mortality and factor of population growth (Chroust, 1984; Dubinský et al., 2010), and communities of *Eimeria* parasites being described extensively in this host species (Forstner and Ilg, 1982; Chroust, 1984; Böckeler et al., 1994; Dubinský et al., 2010; Chroust et al., 2012; Lukešová et al., 2012; Kornaś et al., 2014; Faehndrich et al., 2023). We tested the effects of age, population density, distance to water and rainfall in hares killed by road traffic. We were also interested in whether infection with *Eimeria* spp. may cause clustering of hare–vehicle collisions (HVCs). We addressed this issue by testing the effect of the infection status on the probability of a hare being hit by a vehicle at the hotspot of road mortality.

## Materials and methods

### Field procedures

**Figure 1.**
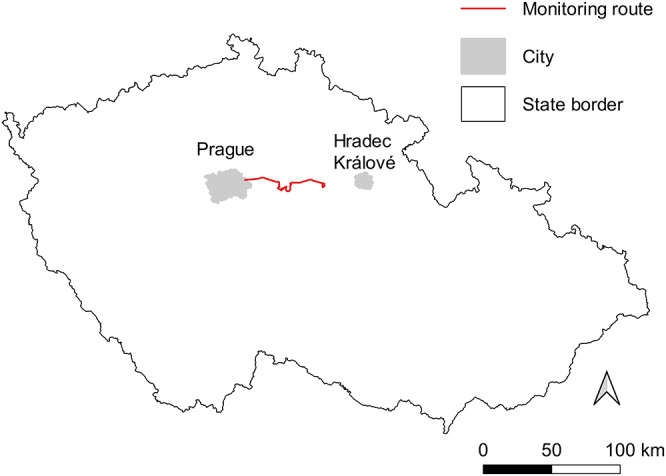
The location of the monitoring route in the Czech Republic where road-killed hares were collected for the analysis of *Eimeria* spp.

A single person (Jan Hušek) searched for the hares killed by the road traffic while driving along an 83.9 km long monitoring route on paved 2-lane roads. The route was located east of the capital of the Czech Republic, Prague (50°5ʹN, 14°26ʹE; Figure 1), in an agricultural landscape composed of conventionally managed fields, mostly cultivated by common crops such as rapeseed, cereals and a low proportion of vegetables interspersed with small to midsize deciduous and coniferous forest patches and urbanized areas. The road surveys were conducted in the morning hours (8:00–10:00 local time) on average every 4.52 (SD = 5.41) days from 11 February 2022 to 24 June 2024 (a total of 192 survey days). When possible, a hare carcass was collected to determine the age and infection with oocysts of *Eimeria* spp. In total, 160 HVCs were detected over the study period, from which 22 whole hare carcasses were collected. All of them were examined for the presence of *Eimeria* spp. oocysts. Age (juvenile/adult) was determined for 17 carcasses; the remaining 5 carcasses could not be aged due to the fatal damage (Table 1).

**Table 1.** Overview of hare carcasses used in this study

			Detected species of *Eimeria*					
Date of finding	GPS	Age	*europaea*	*hungarica*	*leporis*	*robertsoni*	*stefanskii*	*townsendi*
24 Feb. 2022	N50°10ʹ16.14″ E15°16ʹ46.78″	–	Yes			Yes	Yes	
07 Mar. 2022	N50°07ʹ44.26″ E15°06ʹ08.32″	Ad				Yes		
09 Mar. 2022	N50°05ʹ08.92″ E15°05ʹ54.53″	Juv	Yes					Yes
14 Mar. 2022	N50°08ʹ47.54″ E14°50ʹ04.85″	Juv						
15 Mar. 2022	N50°07ʹ17.04″ E15°06ʹ09.86″	Juv						
15 Mar. 2022	N50°06ʹ04.57″ E15°03ʹ47.70″	—						
24 Mar. 2022	N50°07ʹ18.48″ E14°59ʹ06.76″	Ad				Yes		
25 Mar. 2022	N50°07ʹ39.50″ E14°42ʹ25.72″	Ad						
04 Apr. 2022	N50°07ʹ28.88″ E15°06ʹ20.81″	Juv						
08 Apr. 2022	N50°05ʹ12.30″ E15°00ʹ56.38″	Ad	Yes			Yes		
22 Apr. 2022	N50°08ʹ18.71″ E14°54ʹ06.77″	Juv						
18 May 2022	N50°05ʹ11.44″ E15°00ʹ01.26″	—						
03 Mar. 2023	N50°07ʹ15.56″ E14°39ʹ44.96″	Ad			Yes	Yes		
08 Mar. 2023	N50°05ʹ10.26″ E15°00ʹ57.17″	—						
30 Mar. 2023	N50°05ʹ33.22″ E15°06ʹ17.71″	Ad	Yes	Yes				
05 Apr. 2023	N50°06ʹ11.34″ E15°02ʹ48.98″	Juv						
05 Apr. 2023	N50°06ʹ19.91″ E15°03ʹ15.59″	Ad	Yes		Yes			
02 Jun. 2023	N50°09ʹ17.10″ E15°13ʹ21.18″	—	Yes		Yes	Yes		
28 Jun. 2023	N50°04ʹ47.68″ E15°05ʹ08.52″	Juv			Yes	Yes		
02 Aug. 2023	N50°05ʹ54.31″ E15°03ʹ44.82″	Juv	Yes		Yes	Yes		
12 Sep. 2023	N50°05ʹ27.60″ E14°59ʹ03.01″	Ad			Yes			
19 Apr. 2024	N50°08ʹ14.71″ E14°45ʹ53.28″	Ad	Yes			Yes		

The age of hares was determined based on dry eye lens weight following Suchentrunk et al. (1991). Eye lenses were extracted from eye bulbs, which were initially stored in 10% formaldehyde. The lenses were then air-dried at 100 °C for 24 h and weighed. Hares with lenses weighing less than 275 mg were classified as juveniles.

### Identification of *Eimeria* parasites

Fecal samples or a part of the intestine were examined for the presence of oocysts using the standard centrifugation-flotation concentration method with modified Sheather’s sucrose solution of gravity 1.30 followed by light microscopy (Zajac and Conboy, 2006). Samples positive for coccidian oocysts were analysed morphologically and morphometrically using an Olympus BX53 light microscope equipped with a DP-73-1-51 high-resolution image-cooled digital camera, and Olympus cellSens Standard v.1.13 imaging software (Olympus, Tokyo, Japan).

### Explanatory variables

We used spring hunter estimates of hare abundance within a radius of 1 km from each site of HVC as a proxy for hare density. The 1 km radius was considered based on the home range size of the local hare population (Ševčík et al., 2023). Every year on a single day at the end of March, non-professional hunters derive estimates of the size of the spring hare populations using a direct count of all hares seen while walking through their hunting grounds in combination with the local knowledge of hare population from continuous monitoring during previous weeks. The spring hunter estimates of hare abundance from March 2022 were obtained for each hunting territory from the Forestry and Game Management Research Institute. The annual mean abundance was transformed to the density of hares per 100 ha of agricultural land (hereafter referred to as ‘hare density’). Distance between each HVC site and the nearest water course was obtained using QGIS 3.10.1 (QGIS.org, 2025).

Oocysts of *Eimeria* can survive in the environment for several months (Svensson, 1995; Burrell et al., 2020; Bangoura et al., 2022). Hence, we assumed rainfall in the past 3 months (84 days) preceding the date of the carcass finding. The rainfall data for the closest town to each collision site were downloaded from https://www.meteocentrum.cz/archiv-pocasi. The period of 3 months was assessed based on the length of the *Eimeria* life cycle and the high resistance of oocysts in the environment (Svensson, 1995; Pakandl, 2009; Burrell et al., 2020; Mesa-Pineda et al., 2021).

### Statistical analysis

We used logistic regression to test the effect of age (juvenile/adult), population density, distance to the nearest water and rainfall on the probability of infection with *Eimeria* spp. (binary variable: 1 infected/0 uninfected). Due to the low sample size, we tested the effect of each candidate factor in a separate model rather than employing multiple logistic regression.

To identify the hotspots of HVCs, we used a kernel smoothing function from a point pattern within a buffer of 1 km along the monitoring route in QGIS 3.10.1. As a hotspot, we defined kernel densities of HVCs >6. We assigned a location of each hare tested for *Eimeria* infection to whether it was within or outside of one of the hotspots of all HVCs and used logistic regression to test the effect of the infection status (infected/uninfected) on the probability of a sample being from within or outside of an HVC hotspot.

## Results

From the 22 hares analysed, 13 (59.1%) tested positive for *Eimeria* spp. The following 6 species were recorded: *Eimeria europaea, E. hungarica, E. leporis, E. robertsoni, E. stefanskii* and *E. townsendi*. Table 2 presents the prevalences of each species and those reported by other studies from the Czech Republic for comparison. Of the hares tested positive for *Eimeria*, 23.1% were infected with a single species, 53.8% with 2 species and 23.1% with 3 species. The co-infections were the following: *E. robertsoni* + *E. europaea* + *E. leporis* (2 hares), *E. robertsoni* + *E. europaea* + *E. stefanskii* (1), *E. robertsoni* + *E. europaea* (2), *E. robertsoni* + *E. leporis* (2), *E. europaea* + *E. leporis* (1), *E. europaea* + *E. townsendii* (1) and *E. europaea* + *E. hungarica* (1). Original data can be found in Table 1.

**Table 2.** Reported prevalence of *Eimeria* spp. in feces of European hare in the Czech Republic

Species	Prevalence (%)				
	Jirouš (1979)	Chroust (1984)	Pakand (1990)	Lukešová et al. (2012)	This study
*E. babatica*			26.4–31.1	39.5	
*E. cabareti*				64.5	
*E. coquelinae*				68.4	
*E. europaea*	26–55	8.1–10.0	21.2–30.3	34.7	36.4
*E. hungarica*	5	5.0–5.6	6.5–7.6		4.5
*E. leporis*	74–90	37.4–47.1	24.2–45.5	57.8	27.3
*E. macrosculpta*			1.3–2.5	7.3	
*E. orbiculata*				8.3	
*E. pierrcouderti*				15.9	
*E. robertsoni*	74–90	24.8–34.8	36.8–29.4		40.9
*E. sculpta*			6.9–13.4		
*E. semisculpta*	100	3.6–8.3	10.0–15.6		
*E. septentrionalis*		3.4–9.0			
*E. stefanski*				1.8	4.5
*E. townsendi*	5–10	1.5–3.0	67.1–74.8		4.5

**Figure 2.**
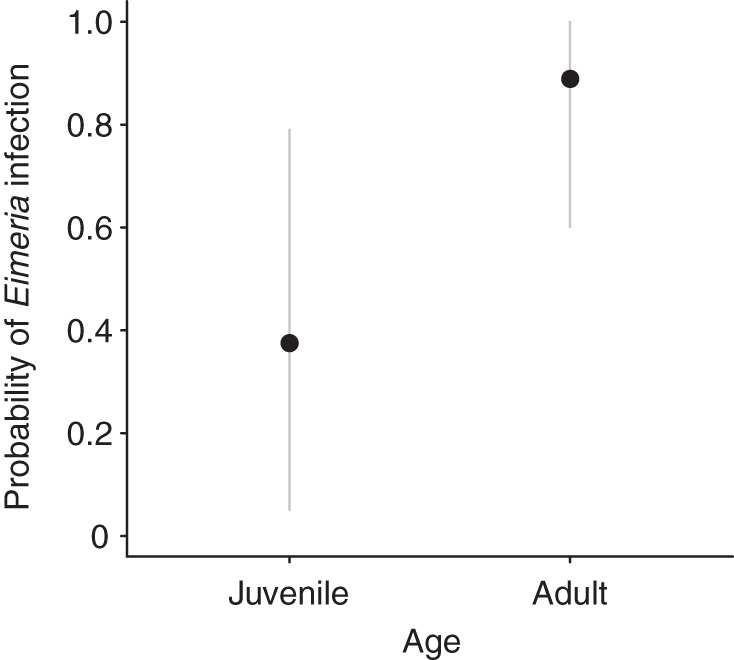
Relationship between age and probability of infection with *Eimeria* spp. in European hare, Czech Republic. The grey lines show 95% confidence intervals.

From the 4 factors tested in separate logistic regression models, only the effect of age was significant (Table 3). The McFadden *R*^2^ index of the model including the effect of age was 0.24, indicating that this factor explained about a quarter of the variability in the data. The model predicted that the probability of *Eimeria* spp. infection was 0.375 (95% CI [0.125, 0.715]) in juveniles and 0.889 (95% CI [0.500, 0.985]), i.e. 2.37× higher, in adults (Figure 2).

**Table 3.** Summary results of the logistic regression models on the effects of age (juvenile/adult), population density, distance to water course and rainfall in the preceding 3 months on the probability of infection by *Eimeria* in European hares in the Czech Republic. Significant effect in bold

Predictor	*β* (s.e.)	Wald	d.f.	*P*	*n*
Model 1					17
Intercept	−0.511 (0.730)	0.49	1	0.484	
**Age_adult_**	**2.590 (1.288)**	**4.00**	**1**	**0.044**	
Model 2					22
Intercept	0.466 (0.873)	0.29	1	0.593	
Density	−0.007 (0.051)	0.02	1	0.896	
Model 3					22
Intercept	1.417 (0.907)	2.40	1	0.118	
Distance to water	−1.011 (0.742)	1.90	1	0.173	
Model 4					22
Intercept	−4.623 (3.000)	2.40	1	0.123	
Rainfall	0.054 (0.034)	2.60	1	0.107	

**Figure 3.**
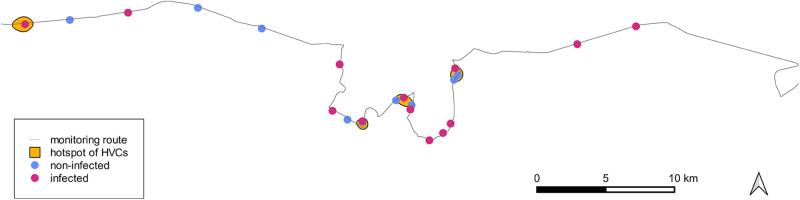
Locations of road-killed hares tested positive (red points) and negative (blue points) for *Eimeria* infection and density hotspots of hare–vehicle collisions (HVCs, orange polygons). Density is the kernel density of a number of collisions on a given road segment during II.2022–VI.2024. The density of HVCs was defined as a hotspot when the number of HVCs is >6.

The probability of a sample being collected from an HVC hotspot was not related to whether an individual tested positive or negative for *Eimeria* spp. (*χ*^2^_1_ = 1.3, *P* = 0.25; Figure 3).

## Discussion

The finding of a positive effect of age on the prevalence of *Eimeria* spp. in European hares was surprising as the opposite has been generally observed among birds and mammals, including leporids (Stodart, 1968; Grès et al., 2003; Pfukenyi et al., 2007; Lassen et al., 2009; Bangoura et al., 2012; Chroust et al., 2012; Rehman et al., 2012; Kornaś et al., 2014; Makau et al., 2017; Faehndrich et al., 2023). The pathogenicity of different *Eimeria* species varies from non-pathogenic to severely pathogenic, the latter often fatal (Pakandl, 2009; Mesa-Pineda et al., 2021; Bangoura et al., 2022). Some *Eimeria* species, especially *E. leporis*, may have caused the deaths of leverets or juveniles early during their life, leaving only more resistant juveniles in the population (Espinosa et al., 2020). A strong negative correlation between the number of oocysts of *E. leporis* and the proportion of juvenile hares in a population has been reported, e.g., from southwestern Slovakia (Dubinský et al., 2010). *E. leporis* had the third highest prevalence in our study, after *E. robertsoni* and *E. europaea.*

Unfortunately, no detailed overview of the pathogenicity of *Eimeria* of European hares has been published that would allow a more critical evaluation (Bangoura and Daugschies, 2018). However, weak and susceptible leverets and juveniles may have also been removed early from the population by mortality factors other than coccidiosis, e.g. predation and/or agricultural operations (Voigt and Siebert, 2020; Cukor et al., 2024). The other tested effects, density, distance to water and rainfall, were not significant predictors of *Eimeria* prevalence. Again, the lack of density effect is surprising, because the positive effect has been generally reported in both domestic and wildlife species (Rodríguez-Vivas et al., 1996; Rehman et al., 2012; Bangoura et al., 2022). Inability to detect the density effect in our study may have been caused by at least 3 aspects. First, hunter estimates may not have approximated the density with a sufficient precision as their reliability may be low (Cukor et al., 2017; Hušek, 2025). Second, assigning each road-kill site with a correct density estimate based on a hunting ground may not be straightforward because about 73% of all road-killed hares were found closer than 500 m from a hunting ground border, at a distance regularly moved by hares from our study population (Ševčík et al., 2023). Third, a decrease in *Eimeria* transmission at low hare densities may have been compensated by a clustered rather than random or regular hare distribution at low population density promoted by a preference for patchy habitat (Kamieniarz et al., 2013; Pavliska et al., 2018).

Relatively large home ranges of hares inhabiting conventional agricultural landscapes may have counteracted the effect of population density on *Eimeria* infections in our study (Ševčík et al., 2023). Also, the sample size used in our study may not have been large enough to detect subtle density effects.

Our study supports a large variation in *Eimeria* prevalences among populations and over the years (Table 2). Yet, *E. europaea, E. leporis* and *E. robertsoni* had high prevalences in all studies from the Czech Republic. Unlike other studies, we did not detect *E. babatica* (Table 2).

We found no relationship between the infection status and the probability of a hare being killed by a vehicle at the hotspot of road mortality. This indicates that infection with *Eimeria* did not contribute to the clustering of HVCs, and it remains for further study whether *Eimeria* may alter levels of hare activity as has been shown in other parasites, including Apicomplexans (McElroy and de Buron, 2014; Horváth et al., 2016; Megía-Palma et al., 2020, 2024).

In conclusion, the positive effect of age on the prevalence of *Eimeria* in European hares does not correspond with the results of a study from the Kraków Province in southern Poland (Kornaś et al., 2014), challenging the generality of a negative effect of age not only in hares but also in other mammals (Craig et al., 2007). Future studies shall shed light on drivers of the spatiotemporal variation in the composition of *Eimeria* parasites in hares.

## Data Availability

The datasets analysed for this study can be found in the manuscript.
